# Euphorbia Corollata

**Published:** 1844-01

**Authors:** 


EUPHORBIA COROLLATA.

We extract the following from a letter of Dr. Preston Capshaw, Madison county, Alabama. From the known activity of most or all the *euphorbiaceæ*, we have supposed this intimation of a new property in the *Corollata* not unworthy of notice. D.

*The Euphorbia Corollata, or Jacob's Root,* has become a common remedy among the populace of this neighborhood, in the cure of Intermittants. The public, and the world, for aught I know, are indebted for the knowledge of its specific virtues, in the above diseases, to a negro by the name of Jacob, who was vending it as a *nostrum*, when I came into the neighborhood four years ago: hence the vulgar name by which it is known to the common people. Jacob, however, did not long enjoy the emoluments arising from the sale of this root. By some means, probably not understood by himself, his secret became known to the people, and has since been extensively used in all the more common forms of intermittant fever.

"Jacob's prescription was a table-spoonful of the powdered root at a single dose, which generally produced vomiting and purging in from one to four hours. Since it has passed into the hands of the public, however, it is seldom pulverised, being prescribed by the *inch*, more or less, according to the diameter of the root, the age of the patient, &c., to be masticated and swallowed after the manner of a raw potatoe, which it is said, in taste, to resemble.

"I am assured by many, who have used it in their families, from one to two years, almost to the exclusion of quinine, that a single dose of it has cured chills, in the majority of cases in which they have given it. When it fails it is given in like manner during the next intermission, &c.

"My personal experience with the article, is not sufficient to warrant an opinion about it, but I incline to the belief, that its remedial virtues have never yet been fully and properly appreciated by the medical profession. It is certainly a very active medicine, promptly and speedily evacuating the stomach and bowels, accompanied by a sensation of warmth through the course of the alimentary canal.

"It has been given in hundreds of cases, and only one has come to my knowledge, in which it appeared to have operated injuriously: this occurred in a case of *grippe*, where it was given in conjunction with tartar emetic, to a child five years old—an almost fatal collapse was the result."

